# Integrative Multidimensional Machine Learning Models for Stroke Prognosis: Age-Stratified and History Engineered Perspectives

**DOI:** 10.3390/diagnostics16091348

**Published:** 2026-04-29

**Authors:** Gawon Lee, Sunyoung Kwon, Seung-Ho Shin, Chulho Kim, Jae Yong Yu

**Affiliations:** 1 Division of DataScience, Hallym University, Chuncheon 24252, Republic of Korea; 2 Data Science for Smart Health (DASH) Laboratory, Hallym University, Chuncheon 24252, Republic of Korea; 3 Healthcare AI assurance Laboratory, Hallym University, Chuncheon 24252, Republic of Korea; 4 Department of Biomedical Informatics, Chuncheon Sacred Heart Hospital, Hallym University College of Medicine, Chuncheon 24253, Republic of Korea; 5 Department of Neurology, Chuncheon Sacred Heart Hospital, Hallym University College of Medicine, Chuncheon 24253, Republic of Korea; 6 Research Institute for Data Science and AI, Hallym University, Chuncheon 24252, Republic of Korea; 7 Department of Emergency Medicine, Hallym University, Chuncheon 24252, Republic of Korea; 8 Department of Population and Quantitative Health Sciences, University of Massachusetts Chan Medical School, Worcester, MA 01655, USA

**Keywords:** stroke, prognosis, multidimensional analysis, machine learning

## Abstract

**Introduction:** Stroke remains a leading cause of mortality and long-term disability worldwide. Accurate prognosis prediction is essential for timely intervention and personalized treatment planning. However, previous studies have often overlooked the role of patients’ medical history, age-specific risk factors, and time-dependent mortality patterns. This study aimed to develop and evaluate machine learning models for predicting mortality in stroke patients by incorporating vital signs, blood test results, demographic characteristics, and medical history, while also exploring subgroup-specific factors. **Methods:** We retrospectively analyzed data from 1780 stroke patients admitted to Hallym University Sacred Heart Hospital between 2018 and 2023. Input features included both original and binarized forms of vital signs and blood test values, along with age and medical history. Random Forest models were developed to predict mortality at 1, 2, and 3 years post-admission, as well as overall mortality. Model performance was assessed using AUC and 95% confidence intervals, and variable importance was evaluated using Mean Decrease Gini and SHAP values. **Results:** The highest predictive performance was observed in a model for patients under 60 using binarized input features, achieving an AUC of 0.995 (CI: 0.98–1). Across all models, pulse rate consistently emerged as the most important predictor. Additional key features included platelet count and diastolic blood pressure. SHAP analysis revealed that pulse rate was associated with higher mortality risk. Subgroup analyses based on age and medical history improved interpretability and predictive power. **Conclusions:** This study demonstrates that integrating clinical indicators with demographic and medical history variables can significantly enhance the accuracy and interpretability of mortality prediction models in stroke patients. The results underscore the importance of stratified modeling and continuous monitoring of vital signs, particularly pulse rate, to support precision stroke care.

## 1. Introduction

Stroke occurs when blood vessels supplying the brain become blocked or rupture, leading to brain damage and lasting effects like hemiplegia, speech impairment, and cognitive dysfunction. According to the World Stroke Organization’s 2022 report, stroke is the second leading cause of death globally, with 12.2 million new cases annually [1,2]. As one in four adults over 25 is at risk, research on prevention, early treatment, and prognosis prediction is crucial. In particular, prognosis prediction research is essential for assessing a patient’s survival probability and establishing an effective treatment plan. With an accurate prediction model, medical professionals can provide personalized treatment for each patient and optimize treatment strategies. The development of a highly reliable prognosis prediction model plays a crucial role in improving the quality of stroke treatment [3,4,5,6,7].

The study published in 2019 investigated patients who experienced acute ischemic stroke for the first time and observed that cerebral arterial stiffness was independently associated with functional decline in acute ischemic stroke patients after three months [8]. Another study published in 2024 in Australia examined the impact of age, gender, and education level on the frequency of healthcare utilization over 12 months. The results identified that age influenced healthcare utilization among middle-aged and older adults with a history of stroke [9]. Age group is an important factor in healthcare research, as it reveals significant differences in patient prognosis [10,11]. A study published in 2025 in the United States analyzed changes in quality of life among patients after hospital discharge following a stroke, at 1-month, 3-month, and 6-month intervals. The results showed a gradual increase in average quality of life scores over time, indicating that patients were recovering toward a relatively stable daily life [12]. Thus, studies with consideration of different outcome periods are important in research as they allow tracking of recovery or deterioration over specific time points [13]. Accurate mortality prediction is critical for guiding clinical decision-making, optimizing resource allocation, and improving long-term patient management in stroke care. However, the complex interactions among demographic characteristics, clinical indicators, and comorbidities make prognosis prediction challenging, highlighting the need for more comprehensive and interpretable modeling approaches. Recent reviews have also noted persistent challenges in stroke machine learning research, including heterogeneity of predictors, limited external validation, and insufficient interpretability [14,15].

While previous studies focused on how highly associated stroke was with other diseases or mortality, few have considered differences in stroke risk and prognosis across age groups [16]. Moreover, most predictive models have been developed based solely on patients’ current health status, often failing to adequately incorporate medical history, which is critical for accurate prognostic assessment. Subgroup analyses including vital signs and blood test results are rare [17], and existing machine learning-based stroke prognosis models often lack interpretability [18], limiting their clinical applicability and reducing their contribution to personalized risk assessment and treatment decisions [19].

To address these limitations, this study aims to develop and evaluate machine learning models for predicting mortality in stroke patients using demographic characteristics, vital signs, blood test results, and medical history. We further investigate whether stratifying patients by age and medical history improves predictive performance and model interpretability. This approach is intended to provide clinically actionable insights for personalized risk assessment and stroke management.

## 2. Materials and Methods

### 2.1. Study Design and Setting

This study is a retrospective study for developing a mortality prediction model from 1780 patients diagnosed with stroke at Hallym University Sacred Heart Hospital between 2018 and 2023. Hallym University Sacred Heart Hospital is a university hospital based in Anyang, Uiwang, and other regions, operating 34 medical departments and employing over 2100 medical professionals. The hospital’s clinical data were sourced from a standardized CDW (Clinical Data Warehouse) which is the HERO (Harmonic intEgrated Research platfOrm) cloud platform, containing high quality electronic health records. The present study protocol was reviewed and approved by the Institutional Review Board of Hallym University Chuncheon Sacred Heart Hospital (IRB No. 2023-09-008). All statistical and machine learning analyses were performed using R software (version 4.4.1; R Foundation for Statistical Computing, Vienna, Austria).

### 2.2. Input Data

The input variables can be categorized into demographic variables, vital signs, blood test results, and categorical versions of these variables. The demographic variables include gender and age. The vital sign variables include body temperature (BT), diastolic blood pressure (DBP), systolic blood pressure (SBP), pulse rate (PR), and respiratory rate (RR). The blood test variables include hemoglobin (HB), hematocrit (HMT), white blood cell count (WBC), platelet count (PC), activated partial thromboplastin time (aPTT), prothrombin time (PT_INR), total cholesterol (TC), blood urea nitrogen (BUN), and creatinine (CRN). Vital sign and blood test variables were binarized based on their normal ranges, classifying values as normal (0) or abnormal (1). The normal reference ranges used for binarization are presented in Table 1.

### 2.3. Feature

To enhance the predictive model, additional binarized variables for age and medical history were created. Age was binarized as 0 for individuals under 60 (age_data_0) and 1 for those aged 60 and above. Medical history variables were binarized to capture prevalent comorbidities. Specifically, the most common conditions were identified based on their frequency in the study population, and the top conditions were selected to construct subgroup variables.

- hist_5 indicates the presence of at least one of hypertension, diabetes, hyperlipidemia, stroke, or heart disease.
- hist_4 indicates the presence of at least one of hypertension, diabetes, stroke, or hyperlipidemia.
- hist_3 indicates the presence of at least one of hypertension, diabetes, or stroke.
- hist_total indicates all considered conditions (hypertension, diabetes, hyperlipidemia, stroke, heart disease, cancer, Hepatitis B, Hepatitis C).

Using these newly generated age and medical history variables, subgroups were defined, allowing for further stratified modeling.

### 2.4. Data Preprocessing

#### 2.4.1. Outlier and Missing Value Handling

Outliers were identified using the standard boxplot method, where values outside 1.5 times the interquartile range (IQR) below the first quartile or above the third quartile were considered extreme. These extreme values were replaced with the median of the corresponding variable to reduce the influence of outliers. If multiple values were recorded for a single variable within one observation, only the first recorded value was retained. Missing values were handled differently depending on the variable type. For binary variables, such as past medical history, missing values were treated as 0, under the assumption that if the information was not recorded, the behavior or condition was not present. For continuous variables, missing values were imputed using the median.

#### 2.4.2. Outcome Variables

In this study, four outcome variables indicating the mortality status of stroke patients were defined to predict the likelihood of death. We utilized national mortality data from Statistics Korea, which includes information such as year of death, region, age group, and disease codes as the cause of death. The outcome variables were created as binary variables representing mortality at specific time points using the admission date. The four outcome variables are as follows:

- outcome_nyear: A binary variable indicating death within *n* year of admission (1 for death, 0 for survival).
- outcome_all: A binary variable indicating the mortality status of the patient, regardless of the time point (1 for death, 0 for survival), which serves as the primary outcome variable for this study.

The *n* above represents 1, 2, and 3, indicating mortality within 1, 2, and 3 years, respectively. Additionally, a predictive model reflecting time-specific mortality was developed using a total of four variables, including an overall mortality variable without specific time constraints.

### 2.5. Machine Learning Models

#### 2.5.1. Machine Learning Models and Evaluation

In this study, a Random Forest model was used to predict the mortality status of stroke patients. Random Forest is an ensemble learning technique that combines multiple decision trees to make final predictions. By aggregating the predictions of each decision tree through a majority voting process, Random Forest helps prevent overfitting and provides high prediction accuracy. Considering these characteristics, it was chosen as the analysis model for this study. The study trained a Random Forest model with 100 trees, and after modeling, the feature importance was checked to identify the key variables that influence mortality. To further reduce the risk of overfitting and to ensure the robustness of the results, we performed 10-fold cross-validation. The dataset was randomly partitioned into 10 equal subsets, with nine subsets used for training and one for validation in each iteration. This process was repeated 10 times, and model performance was reported as the average across all folds.

#### 2.5.2. Modeling Procedure

Since prediction results may vary depending on the combination of input variables, various combinations of independent variables were constructed to train the model. The combinations of independent variables are as follows:

1. Original vital signs and blood test values (*v*/*o*, *b*/*o*);
2. Binary-encoded vital signs and original blood test values (*v*/*b*, *b*/*o*);
3. Original vital signs and binary-encoded blood test values (*v*/*o*, *b*/*b*);
4. Binary-encoded vital signs and blood test values (*v*/*b*, *b*/*b*).

Original refers to the actual measured vital signs and blood test values, while binary indicates whether these values fall within the normal range, expressed as 0 or 1. Accordingly, the abbreviations for each variable combination follow these rules: ‘*v*/*o*’ represents original vital signs, while ‘*v*/*b*’ denotes binary vital signs. Similarly, ‘*b*/*o*’ refers to original blood test values, whereas ‘*b*/*b*’ signifies binary blood test values. Using these combinations of variables, the model was built with 70% of the data used as the training set and the remaining 30% used as the test set for model validation.

#### 2.5.3. Model Evaluation Metrics

Model performance was evaluated using accuracy, the area under the curve (AUC), and the 95% confidence interval (CI) as key metrics. Accuracy indicates the proportion of predictions that match the actual data, while AUC evaluates the overall performance of the model based on the area under the receiver operating characteristic (ROC) curve.

Sensitivity and specificity were defined as follows:

$$Sensitivity=\frac{TP}{TP+FN}$$

$$Specificity=\frac{TN}{TN+FP}$$

where *TP*, *TN*, *FP*, and *FN* represent true positives, true negatives, false positives, and false negatives, respectively.

AUC represents the area under the ROC curve, which plots the true positive rate (sensitivity) against the false positive rate (1-specificity) at various threshold settings. A higher AUC indicates better discrimination between mortality and non-mortality cases.

A bar chart was created to compare the AUC values and 95% confidence intervals of each model, allowing identification of the best-performing model and the optimal combination of independent variables. The analysis was performed using R software, version 4.4.1.

### 2.6. Model Explainability

Model interpretability was assessed using two complementary approaches: feature importance based on Mean Decrease Gini and SHAP analysis. Feature importance was first calculated separately for each Random Forest model based on the extent to which each variable reduced Gini impurity across trees [20]. To provide an overall summary across model configurations, feature importance values were additionally averaged across relevant subgroup models within each input variable format, and the variable with the highest average importance was reported in the main manuscript. In the Supplementary Tables S1–S3, only the single top-ranked feature for each model was presented to provide a concise model-level summary. SHAP values were calculated to quantify each variable’s contribution to the prediction [21], and SHAP analysis was performed only for the best-performing representative model to visually illustrate the contribution and direction of predictors within that model.

### 2.7. Statistical Analysis

Categorical variables were expressed as frequency and percentages; continuous variables were expressed as means and standard deviations. Comparison tests for each outcome were performed with *t*-test and chi-square tests at 5% significance levels.

**Figure 1:**
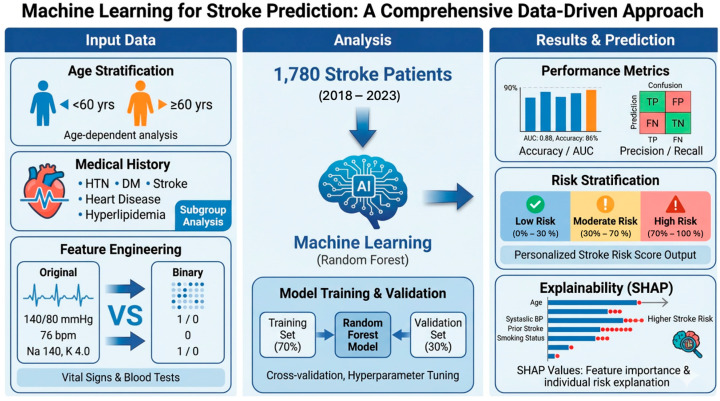
Overview of the study design. This study aimed to develop Random Forest models to predict mortality in stroke patients using demographic data, medical history, vital signs, and blood test results, and to evaluate model performance using AUC and SHAP values. Arrows indicate the analytical workflow, colors denote risk levels and prediction outcomes, and dots in the SHAP plot represent individual samples.

Figure 1 shows the overall study design of our study. The input variables included age, gender, the presence of the top 5, 4, or 3 medical history variables, the original values of vital signs and blood test results, and their corresponding binarized forms. Age and medical history were used for subgroup analyses, and various model combinations were constructed based on the format of vital signs and blood test results (continuous or binary). The output data comprised four outcomes: mortality within 1, 2, and 3 years, and all-cause mortality. Random Forest models were developed using the input and output variables, and model performance was assessed by comparing AUC values. Variable importance was evaluated using both feature importance scores and SHAP values.

## 3. Results

Baseline demographic and clinical characteristics of the study population, stratified by mortality status, are presented in Table 2. In terms of age, the proportion of individuals aged 60 and above was significantly higher in the mortality group (92.1%) compared to the non-mortality group (81.6%). Regarding vital signs, the pulse rate was significantly higher (82.1 ± 15.0) in the mortality group than in the non-mortality group. In blood tests, hemoglobin (13.7 ± 1.9), hematocrit (40.3 ± 5.1), platelet count (221.2 ± 54.2), and total cholesterol (172.9 ± 40.1) were significantly higher in the non-mortality group than in the mortality group.

**Figure 2:**
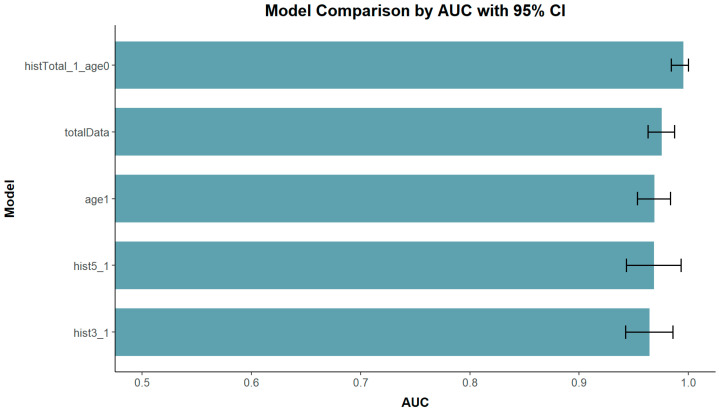
Graph of AUC values and 95% confidence intervals for various models, assessing their overall predictive performance. These models were selected to represent subgroup-specific patterns and provide a concise summary of key findings. Each representative model was selected from each subgroup category (total data, age-stratified, history-stratified, and combined age- and history-stratified models), and in each case, the model with the highest AUC within that category was chosen. Models with an AUC of 1 were not displayed in the figure to avoid potential overfitting concerns. histTotal_1_age0: All history variables were binarized to 1, and age was coded as 0 for individuals under 60. totalData: The complete dataset including all variables. age1: Age was binarized as 1 for individuals aged 60 and above. hist5_1: History variable was binarized into 1 for the top 5. hist3_1: History variable was binarized into 1 for the top 3. Results for all model combinations across all outcomes are provided in Supplementary Table S4.

As shown in Figure 2, the histTotal_1_age0 model showed the highest AUC value of 0.995 (95%, CI: 0.983–1) indicating the best predictive performance. This was followed by the totalData model with an AUC of 0.975 (95%, CI: 0.963–0.987), and the age1 model with 0.968 (95%, CI: 0.953–0.983), both demonstrating strong predictive abilities. The hist_5 and hist_3 models followed with AUC values of 0.968 (95%, CI: 0.943–0.993) and 0.964 (95%, CI: 0.942–0.985). These five models were selected as representative high-performing models to highlight the main subgroup specific findings. Full results for all model combinations are provided in Supplementary Table S4.

As shown in Table 3, In the vital_original model, pulse rate showed the highest im-portance with a value of 23. In the vital_binary model, DBP_class emerged as the most important variable with an importance of 3.89. In the blood_original and blood_binary models, platelet count and White blood cell_class recorded the highest importance values of 13 and 6.11, respectively. In the vital_original_blood_original and vital_original_blood_binary models, pulse rate was identified as the most important variable with importance values of 9.41 and 16.5, respectively. In the vital_binary_blood_original model, platelet count had the highest importance at 10.9, while in the vital_binary_blood_binary model, DBP_class was the top variable with an importance of 7.04.

**Figure 3:**
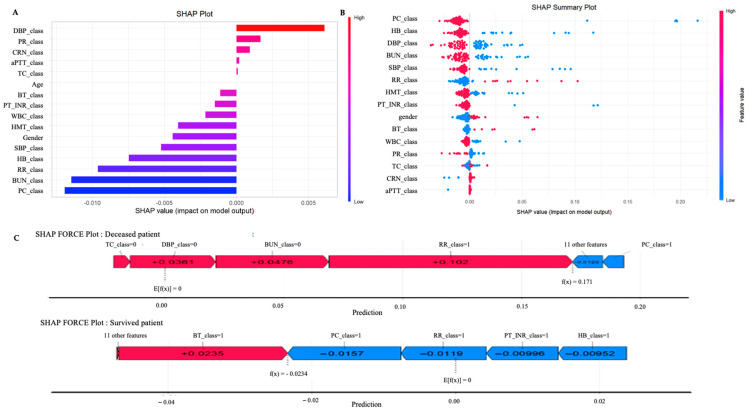
SHAP analysis for the best-performing model (histTotal_1_age0 with outcome_all), visualizing the contribution of each variable to mortality prediction. (**A**) shows the overall contribution of each feature across all patients in the representative model; (**B**) shows the distribution of SHAP values for each feature across all patients in that model; (**C**) presents SHAP force plots for a randomly selected deceased patient (upper) and survived patient (lower). Variables with the ‘class’ indicate binarized versions of the original variables. DBP: diastolic blood pressure; aPTT: activated partial thromboplastin time; WBC: white blood cell; CRN: creatinine; PR: pulse rate; BUN: blood urea nitrogen; BT: body temperature: PT: prothrombin time; INR: International normalized ratio; HMT: hematocrit; SBP: systolic blood pressure; PC: platelet count; HB: hemoglobin; TC: total cholesterol; RR: respiratory rate.

Figure 3A presents the SHAP analysis for the best-performing model, providing a model-specific visualization of how individual predictors contributed to mortality prediction. In this representative model, DBP_class showed the highest positive SHAP value (0.006), followed by PR_class (0.001) and CRN_class (0.0009). In contrast, PC_class (−0.0119) and BUN_class (−0.0114) showed the most negative SHAP values, while RR_class and HB_class also demonstrated negative contributions. Figure 3B further illustrates the distribution of these SHAP values across patients within the same model. PC_class showed the widest distribution, with high feature values (red) concentrated on the negative side and low feature values (blue) extending to the positive side. RR_class and PT_INR_class showed notable positive SHAP values, while DBP_class and BUN_class displayed broad distributions across both positive and negative directions. To illustrate how these features contribute at the individual patient level, Figure 3C presents SHAP force plots for randomly selected deceased and survived patients. In the deceased patient, RR_class showed the largest positive contribution (+0.102), followed by BUN_class (+0.0476) and DBP_class (+0.0361), while PC_class and 11 other features exerted negative contributions, resulting in a final predicted probability of 0.171. In contrast, the survived patient showed a negative predicted value of −0.0234, indicating a below-average probability of mortality, with BT_class as the only positive contributor (+0.0235).

## 4. Discussion

This study aimed to develop a predictive model for mortality risk in stroke patients. The dataset was divided into subgroups based on patients’ age and medical history, and a Random Forest algorithm was used for model training. In the best-performing subgroup model, histTotal_1_age0, which used binarized variables in patients younger than 60 years with at least one medical history condition, variables related to platelet count and systolic blood pressure showed high model-level importance. SHAP analysis was additionally used to visually illustrate the contribution and direction of predictors within this representative model. These findings suggest that the use of binarized variables may improve predictive performance while also enhancing interpretability in clinical settings.

Previous machine learning studies in stroke and cardiovascular risk prediction have identified general predictors across entire populations [22,23,24] but have largely overlooked subgroup-specific differences. Our study addresses this gap by stratifying patients based on medical history, revealing distinct predictors within subgroups. This approach enhances model interpretability and provides clinically actionable insights that are not apparent in analyses of the overall population.

In patients younger than 60 with at least one comorbidity, whether vital signs and laboratory results fell within the normal range significantly influenced prognostic prediction. To enhance clinical interpretability and account for potential non-linear associations, all continuous variables were converted into binary indicators representing whether each value was within the normal range. These binarized variables were incorporated into the modeling process, which contributed to dimensionality reduction, helped stabilize model training, and improved overall performance. As a result, the model incorporating these factors achieved the highest AUROC. When analyzing subgroup models that incorporate medical history stratification (including models combined with age stratification, hist3_1 model, hist4_1 model, hist5_1 model, histTotal model, and their age-stratified counterparts, as shown in Supplementary Tables S4 and S5), systolic blood pressure and pulse rate consistently emerged as the most influential variables. This finding is consistent with previous studies, showing that low prestroke SBP (<120 mm Hg) in patients with at least one comorbidity is associated with higher post-stroke mortality risk [25], that laboratory-based features such as hemoglobin, HDL, and creatinine are important predictors of stroke recurrence [14], and that Random Forest modeling effectively predicts long-term mortality and morbidity in stroke patients [15]. Our study extends these findings by showing that these vital signs and lab variables have predictive value specific to subgroups, particularly in younger patients with comorbidities, highlighting the importance of stratified analysis.

Pulse rate emerged as the significant predictor across nearly all models for stroke outcomes. This finding is consistent with a U.S. cohort study reporting that higher heart rate during the acute phase of ischemic stroke predicted recurrent stroke, myocardial infarction, and death within one year [26,27]. Similarly, prior work has shown that elevated resting heart rate is associated with increased post stroke mortality [28], and a secondary rise in heart rate during the acute phase has been reported as an early indicator of fatal outcomes [29]. Importantly, our subgroup analyses reveal that pulse rate remains a strong prognostic factor even when focusing on specific patient groups, highlighting its clinical relevance for monitoring and early intervention in stroke patients. Pulse rate could inform clinical decision-making, helping identify patients who require closer monitoring, early cardiovascular evaluation, or individualized interventions such as beta blocker adjustment or targeted fluid management. Integrating pulse rate trends into alert systems may enable rapid identification of high-risk patients, supporting timely escalation of care and personalized treatment planning.

Feature importance analysis and SHAP analysis served complementary roles in interpreting the best-performing model. Feature importance highlighted variables related to platelet count and systolic blood pressure as influential predictors at the model level, whereas SHAP analysis visually demonstrated how predictors such as PR, PC, BUN, RR, and HB contributed to mortality prediction within the representative model. Rather than providing a single unified ranking across all analyses, these approaches were intended to offer complementary perspectives on model interpretability. Elevated resting PR was associated with increased mortality risk, in line with previous large cohort studies showing higher stroke risk with increased baseline heart rate [30,31]. PC showed a notable impact on predictions, aligning with findings that both high and low platelet counts within the normal range are linked to worse outcomes [32,33]. BUN also contributed to mortality risk, reflecting renal and metabolic status, with prior studies showing a non-linear relationship with poor outcomes [34]. Beyond their statistical importance, these variables can be leveraged clinically to stratify patients into high-risk subgroups that may not be apparent using standard assessments. Patients exhibiting simultaneous elevations in PR and BUN or abnormal PC values could be prioritized for intensified monitoring, early intervention, or tailored therapies such as optimization of cardiovascular management, careful fluid balance, or personalized anticoagulation strategies. This integrative data-driven approach enables proactive prioritization of clinical resources and facilitates individualized care plans, illustrating how interpretable machine learning can translate predictive insights into actionable decisions.

Being in the abnormal range of DBP was also associated with an increased probability of mortality. This observation is consistent with previous clinical studies showing that elevated diastolic blood pressure is linked to higher stroke incidence and mortality. For instance, one study reported that each 1 mmHg increase in diastolic blood pressure corresponded to an approximately 3% increase in stroke-related mortality [35]. Moreover, in patients with complete middle cerebral artery stroke, higher diastolic blood pressure at admission was independently associated with in-hospital mortality. In this cohort, the median diastolic blood pressure was 90 mmHg in patients who died versus 80 mmHg in survivors, and the risk of death increased by 5% for each mmHg increase in diastolic blood pressure, even after adjusting for other risk factors [36]. These findings highlight the clinical relevance of diastolic blood pressure for early intervention and patient management. They also suggest the potential to develop personalized strategies for stabilizing diastolic blood pressure in order to optimize outcomes.

This study presents several meaningful findings that distinguish it from previous research; however, it has certain limitations. First, as this study is based on retrospective data analysis, the criteria for patient selection are not clearly defined, which may limit its representativeness. Additionally, missing or inaccurate information in medical records is a potential concern. Second, since the analysis is based on data from a single institution, generalizing the findings to other hospitals, regions, or populations may be challenging. Third, the absence of resampling techniques to address class imbalance may have influenced model performance. Finally, comparisons with other machine learning algorithms, such as logistic regression and XGBoost, were not performed, which may have limited the comprehensiveness of the evaluation. This study intentionally focused on isolating the effects of data characteristics and subgroup-specific features. Given these limitations, future research should incorporate data from multiple institutions and adopt a prospective study design, along with external validation using independent datasets, to ensure a more systematic and reliable analysis. Moreover, multicenter studies with larger sample sizes could enable imputation of missing data that accounts for variability across institutions, enhancing the reliability of the analysis. Future studies should also consider the application of resampling techniques, such as SMOTE, to address class imbalance, as well as comparative analyses across various machine learning algorithms to assess the performance and robustness of the proposed model. In addition, future research should consider potential confounders, incorporate imaging and genetic data, and explore the integration of predictive models into clinical decision-support systems to further enhance clinical utility.

## 5. Conclusions

This study demonstrates that stratified machine learning analyses based on patients’ age and medical history can identify subgroup specific predictors of stroke mortality, providing more clinically actionable insights than conventional models. In the model with the highest predictive performance, pulse rate and blood pressure emerged as key prognostic factors in patients younger than 60 with at least one medical history condition. These findings can inform personalized care strategies. Future work should focus on multicenter validation and integration into real world clinical workflows to ensure generalizability and maximize impact on patient outcomes.

## Figures and Tables

**Table 1:** Normal reference ranges used for binarization of clinical variables.

Feature Name	Lower	Upper
Body temperature, °C	36	37.5
Diastolic blood pressure, mmHg	60	80
Systolic blood pressure, mmHg	90	120
Pulse rate, beats per minute	50	100
Respiratory rate, beats per minute	12	20
Hemoglobin, g/dL	12	18
Hematocrit, %	37	50
White blood cell count, 10^9^/L	4.0	10.0
Platelet count, 10^9^/L	130	450
Activated partial thromboplastin time, seconds	25	45
Prothrombin time, seconds	0.8	1.2
Total cholesterol, mg/dL	140	200
Blood urea nitrogen, mg/dL	10	26
Creatinine, mg/dL	0.5	1.4

**Table 2:** Baseline demographic and clinical characteristics stratified by mortality status.

Variables	Non-Mortality (*n* = 1628)	Mortality (*n* = 152)	*p*-Value
Gender, *n* (%)			0.104
- Male	952 (58.5%)	78 (51.3%)	
- Female	676 (41.5%)	74 (48.7%)	
Age, *n* (%)			0.002
- Under 60 s	299 (18.4%)	12 (7.9%)	
- 60 s and above	1329 (81.6%)	140 (92.1%)	
Vital sign, mean ± SD			
- Body temperature, °C	36.7 ± 0.4	36.6 ± 0.4	0.519
- Diastolic blood pressure, mmHg	82.0 ± 11.8	80.9 ± 11.5	0.286
- Systolic blood pressure, mmHg	140.5 ± 20.7	138.0 ± 22.6	0.164
- Pulse rate, bpm	76.5 ± 12.8	82.1 ± 15.0	0.000
- Respiratory rate, bpm	19.3 ± 1.6	19.3 ± 1.8	0.881
Blood test, mean ± SD			
- Hemoglobin, g/dL	13.7 ± 1.9	12.6 ± 1.8	<0.001
- Hematocrit, %	40.3 ± 5.1	37.6 ± 5.4	<0.001
- WBC, 10^3^/μL	7.5 ± 2.2	7.4 ± 2.4	0.597
- Platelet count, 10^3^/μL	221.2 ± 54.2	199.3 ± 60.1	<0.001
- Activated partial thromboplastin time, seconds	29.8 ± 3.2	29.0 ± 3.2	0.006
- Prothrombin time—INR, seconds	1.0 ± 0.1	1.0 ± 0.1	<0.001
- Total cholesterol, mg/dL	172.9 ± 40.1	154.7 ± 43.9	<0.001
- Blood urea nitrogen, mg/dL	14.3 ± 5.8	14.7 ± 5.5	0.444
- Creatinine, mg/dL	0.9 ± 0.2	0.9 ± 0.2	0.093

INR: International normalized ratio; WBC: white blood cell count; SD: standard deviation; *p*-value were calculated with *t*-test for continuous variable and chi-square test for categorical variable.

**Table 3:** Highest average feature importance based on Mean Decrease Gini across each input variable format.

Input Variable Format	Variable with the Highest Average	Importance (Mean Decrease Gini)
vital_original	Pulse rate	23.00
vital_binary	DBP_class	3.89
blood_original	Platelet count	13.00
blood_binary	WBC_class	6.11
vital_original_blood_original	Pulse rate	9.41
vital_original_blood_binary	Pulse rate	16.50
vital_binary_blood_original	Platelet count	10.90
vital_binary_blood_binary	DBP_class	7.04

Variables labeled with ‘class’ indicate binarized versions of the original variables. The feature importance values shown in Table 3 represent the average Mean Decrease Gini calculated across all relevant subgroup models for each input variable format. For each format, the variable with the highest average importance was identified and reported. Table 3 reflects variables that were consistently influential across models within each format, rather than the top predictor from each model. DBP: diastolic blood pressure; WBC: white blood cell.

## Data Availability

The data presented in this study are available on request from the corresponding author due to the privacy issue of patient data.

## Supplementary Materials

The following supporting information can be downloaded at: https://www.mdpi.com/article/10.3390/diagnostics16091348/s1, Table S1: Demographic table summarizes the differences in key clinical variables between the mortality within 1 year and non-mortality groups. Table S2: Demographic table summarizes the differences in key clinical variables between the mortality within 2 year and non-mortality groups Table S3: Demographic table summarizes the differences in key clinical variables between the mortality within 3 year and non-mortality groups. Table S4: AUC values and 95% confidence intervals for various models, assessing their overall predictive performance. Table S5: Top-ranked feature importance result for each model. For each combination of subgroup, outcome, and input variable format, only the single variable with the highest feature importance is shown as a concise summary; the full set of feature importance values for all variables is not presented in this table. Table S6: Number of patients in subgroups defined by age and medical history.

## Abbreviations

The following abbreviations are used in this manuscript:

BT	Body temperature
DBP	Diastolic blood pressure
SBP	Systolic blood pressure
PR	Pulse rate
RR	Respiratory rate
HB	Hemoglobin
HMT	Hematocrit
WBC	White blood cell count
PC	Platelet count
aPTT	Activated partial thromboplastin time
PT_INR	Prothrombin time
TC	Total cholesterol
BUN	Blood urea nitrogen
CRN	Creatinine
AUC	Area under the curve
CI	Confidence interval
AUROC	Area under receiver operating curve
AUPRC	Area under precision-recall curve

## References

[1] Feigin V.L., Brainin M., Norrving B., Martins S.O., Pandian J., Lindsay P., Grupper M.F., Rautalin I. (2025). World Stroke Organization: Global Stroke Fact Sheet 2025. Int. J. Stroke.

[2] Towfighi A., Saver J.L. (2011). Stroke declines from third to fourth leading cause of death in the United States: Historical perspective and challenges ahead. Stroke.

[3] Gao M.M., Wang J., Saposnik G. (2020). The art and science of stroke outcome prognostication. Stroke.

[4] Abujaber A.A., Albalkhi I., Imam Y., Yaseen S., Nashwan A.J., Akhtar N., Alkhawaldeh I.M. (2025). Machine learning-based prediction of 90-day prognosis and in-hospital mortality in hemorrhagic stroke patients. Sci. Rep..

[5] Klug J., Leclerc G., Dirren E., Carrera E. (2024). Machine learning for early dynamic prediction of functional outcome after stroke. Commun. Med..

[6] Goh B., Bhaskar S.M. (2024). Evaluating machine learning models for stroke prognosis and prediction in atrial fibrillation patients: A comprehensive meta-analysis. Diagnostics.

[7] Irie F., Matsumoto K., Matsuo R., Nohara Y., Wakisaka Y., Ago T., Nakashima N., Kitazono T., Kamouchi M. (2024). Predictive performance of machine learning–based models for poststroke clinical outcomes in comparison with conventional prognostic scores: Multicenter, hospital-based observational study. JMIR AI.

[8] Fu X., Chu C., Li X., Gao Q., Jia J. (2019). Cerebral arterial stiffness for predicting functional outcome in acute ischemic stroke. Hypertens. Res..

[9] Sibbritt D., Bayes J., Peng W., Adams J. (2024). Demographic factors effect stroke-related healthcare utilisation among Australian stroke survivors. Sci. Rep..

[10] Meng L., Thapa R., Delgado M.G., Gomez M.F., Ji R., Knepper T.C., Hubbard J.M., Wang X., Permuth J.B., Kim R.D. (2023). Association of age with treatment-related adverse events and survival in patients with metastatic colorectal cancer. JAMA Netw. Open.

[11] Li K., Chadha M., Moshier E., Rosenstein B.S., Jandu H.K., Veal C.D., Fachal L., Luccarini C., Aguado-Barrera M.E., Altabas M. (2025). Age-stratified analysis of health-related quality of life in patients with early-stage breast cancer receiving adjuvant radiation therapy and endocrine therapy. J. Geriatr. Oncol..

[12] Butsing N., Voss J.G., Keandoungchun J., Thongniran N., Griffin M.T.Q. (2025). Changes of health-related quality of life within 6 months after stroke by clinical and sociodemographic factors. Sci. Rep..

[13] Shin S., Lee Y., Chang W.H., Sohn M.K., Lee J., Kim D.Y., Shin Y.-I., Oh G.-J., Lee Y.-S., Joo M.C. (2022). Multifaceted assessment of functional outcomes in survivors of first-time stroke. JAMA Netw. Open.

[14] Schwartz L., Anteby R., Klang E., Soffer S. (2023). Stroke mortality prediction using machine learning: Systematic review. J. Neurol. Sci..

[15] Shurrab S., Guerra-Manzanares A., Magid A., Piechowski-Jozwiak B., Atashzar S.F., Shamout F.E. (2024). Multimodal machine learning for stroke prognosis and diagnosis: A systematic review. IEEE J. Biomed. Health Inform..

[16] Santos L.I., Camargos M.O., D’Angelo M.F.S.V., Mendes J.B., De Medeiros E.E.C., Guimarães A.L.S., Palhares R.M. (2022). Decision tree and artificial immune systems for stroke prediction in imbalanced data. Expert. Syst. Appl..

[17] Debette S., Markus H.S. (2022). Stroke genetics: Discovery, insight into mechanisms, and clinical perspectives. Circ. Res..

[18] Abedi V., Avula V., Chaudhary D., Shahjouei S., Khan A., Griessenauer C.J., Li J., Zand R. (2021). Prediction of long-term stroke recurrence using machine learning models. J. Clin. Med..

[19] Chen M., Qian D., Wang Y., An J., Meng K., Xu S., Liu S., Sun M., Li M., Pang C. (2024). Systematic review of machine learning applied to the secondary prevention of ischemic stroke. J. Med. Syst..

[20] Fernandez-Lozano C., Hervella P., Mato-Abad V., Rodríguez-Yáñez M., Suárez-Garaboa S., López-Dequidt I., Estany-Gestal A., Sobrino T., Campos F., Castillo J. (2021). Random forest-based prediction of stroke outcome. Sci. Rep..

[21] Prendin F., Pavan J., Cappon G., Del Favero S., Sparacino G., Facchinetti A. (2023). The importance of interpreting machine learning models for blood glucose prediction in diabetes: An analysis using SHAP. Sci. Rep..

[22] Sailasya G., Kumari G.L.A. (2021). Analyzing the performance of stroke prediction using ML classification algorithms. Int. J. Adv. Comput. Sci. Appl..

[23] Ivanov I.G., Kumchev Y., Hooper V.J. (2023). An optimization precise model of stroke data to improve stroke prediction. Algorithms.

[24] Vinholt P.J., Hvas A.-M., Frederiksen H., Bathum L., Jørgensen M.K., Nybo M. (2016). Platelet count is associated with cardiovascular disease, cancer and mortality: A population-based cohort study. Thromb. Res..

[25] Aparicio H.J., Tarko L.M., Gagnon D., Costa L., Galloway A., Demissie S., Djousse L., Seshadri S., Cho K., Wilson P.W.F. (2022). Low Blood Pressure, Comorbidities, and Ischemic Stroke Mortality in US Veterans. Stroke.

[26] Lee K.J., Kim B.J., Han M.K., Kim J.T., Choi K.H., Shin D.I., Cha J.K., Kim D.H., Kim D.E., Ryu W.S. (2022). Effect of heart rate on 1-year outcome for patients with acute ischemic stroke. J. Am. Heart Assoc..

[27] Lee J.-D., Kuo Y.-W., Lee C.-P., Huang Y.-C., Lee M., Lee T.-H. (2022). Initial in-hospital heart rate is associated with long-term survival in patients with acute ischemic stroke. Clin. Res. Cardiol..

[28] Heuer C., Gebhard C., Shuaib A., Held U., Wegener S., VISTA collaborators (2024). High resting heart rate is associated with cardiovascular death in patients with stroke, independent of sex. Cerebrovasc. Dis..

[29] Kallmünzer B., Bobinger T., Kopp M., Kurka N., Arnold M., Hilz M.-J., Schwab S., Köhrmann M. (2015). Impact of heart rate dynamics on mortality in the early phase after ischemic stroke: A prospective observational trial. J. Stroke Cerebrovasc. Dis..

[30] O’Neal W.T., Qureshi W.T., Judd S.E., Meschia J.F., Howard V.J., Howard G., Soliman E.Z. (2015). Heart rate and ischemic stroke: The REasons for Geographic And Racial Differences in Stroke (REGARDS) study. Int. J. Stroke.

[31] Zhang D., Shen X., Qi X. (2016). Resting heart rate and all-cause and cardiovascular mortality in the general population: A meta-analysis. CMAJ.

[32] Yang M., Pan Y., Li Z., Yan H., Zhao X., Liu L., Jing J., Meng X., Wang Y., Wang Y. (2019). Platelet count predicts adverse clinical outcomes after ischemic stroke or TIA: Subgroup analysis of CNSR II. Front. Neurol..

[33] Kanazawa K., Miyamoto N., Hira K., Kijima C., Ueno Y., Hattori N. (2022). Baseline platelet count may predict short-term functional outcome of cerebral infarction. BMC Neurol..

[34] Zhou P., Liu R.L., Wang F.X., Hu H.F., Deng Z. (2024). Blood urea nitrogen has a nonlinear association with 3-month outcomes with acute ischemic stroke: A second analysis based on a prospective cohort study. Clin. Nutr. ESPEN.

[35] Palmer A.J., Bulpitt C.J., Fletcher A.E., Beevers D.G., Coles E.C., Ledingham J.G., O’Riordan P.W., Petrie J.C., Rajagopalan B.E., Webster J. (1992). Relation between blood pressure and stroke mortality. Hypertension.

[36] Caso V., Agnelli G., Alberti A., Venti M., Acciarresi M., Palmerini F., Paciaroni M. (2012). High diastolic blood pressure is a risk factor for in-hospital mortality in complete MCA stroke patients. Neurol. Sci..

