# Supplementary material for: Integrative Multidimensional Machine Learning Models for Stroke Prognosis: Age-Stratified and History Engineered Perspectives
Source: Diagnostics (Basel). 2026 Apr 29;16(9):1348. doi: 10.3390/diagnostics16091348 (PMC13163306; doi:10.3390/diagnostics16091348)
Supplement: Supplementary file 1 [file diagnostics-16-01348-s001.zip › diagnostics-4190237-supplementary.pdf]

Supplementary Table S1. Demographic table summarizes the differences in key clinical variables between the mortality within 1 year and non-mortality groups.

Variables	Non-mortality (n=1667)	Mortality (n=113)	p-value
Gender, n (%)			0.175
Male	972 (58.3%)	58 (51.3%)	
Female	695 (41.7%)	55 (48.7%)	
Age, n (%)			0.018
Under 60s	301 (18.1%)	10 (8.8%)	
60s and above	1366 (81.9%)	103 (91.2%)	
Vital sign, mean ± SD			
Body temperature, °C	37.7 ± 0.4	36.7 ± 0.4	0.922
Diastolic blood pressure, mmHg	82.0 ± 11.8	81.3 ± 11.4	0.547
Systolic blood pressure, mmHg	140.5 ± 20.8	137.7 ± 21.5	0.169
Pulse rate, bpm	76.7 ± 12.8	82.4 ± 15.4	< 0.001
Respiratory rate, bpm	19.3 ± 1.6	19.4 ± 1.9	0.579
Blood test, mean ± SD			
Hemoglobin, g/dL	13.6 ± 1.9	12.6 ± 1.9	< 0.001
Hematocrit, %	40.2 ± 5.1	37.7 ± 5.5	< 0.001
WBC Count, $10^3/\mu L$	7.5 ± 2.2	7.4 ± 2.5	0.583
Platelet count, $10^3/\mu L$	221.0 ± 54.2	193.7 ± 60.5	< 0.001
Activated partial thromboplastin time, seconds	29.7 ± 3.2	29.1 ± 3.4	0.055
Prothrombin time – INR, seconds	1.0 ± 0.1	1.1 ± 0.1	< 0.001
Total cholesterol, mg/dL	172.7 ± 40.2	152.1 ± 43.9	< 0.001
Blood urea nitrogen, mg/dL	14.3 ± 5.8	14.7 ± 5.5	0.422
Creatinine, mg/dL	0.9 ± 0.2	0.9 ± 0.2	0.068

INR: International normalized ratio; WBC: White blood cell count; SD: standard deviation; p-value were calculated with t-test for continuous variable and chi-square test for categorical variable.

Supplementary Table S2. Demographic table summarizes the differences in key clinical variables between the mortality within 2 year and non-mortality groups.

Variables	Non-mortality (n=1650)	Mortality (n=130)	p-value
Gender, n (%)			0.154
Male	963 (58.4%)	67 (51.5%)	
Female	687 (41.6%)	63 (48.5%)	
Age, n (%)			0.003
Under 60s	301 (18.2%)	10 (7.7%)	
60s and above	1349 (81.8%)	120 (92.3%)	
Vital sign, mean ± SD			
Body temperature, °C	36.7 ± 0.4	36.6 ± 0.4	0.771
Diastolic blood pressure, mmHg	82.0 ± 11.8	81.3 ± 11.4	0.533
Systolic blood pressure, mmHg	140.4 ± 20.7	138.5 ± 22.5	0.313
Pulse rate, bpm	76.6 ± 12.8	82.1 ± 15.4	< 0.001
Respiratory rate, bpm	19.3 ± 1.6	19.2 ± 1.9	0.646
Blood test, mean ± SD			
Hemoglobin, g/dL	13.6 ± 1.9	12.6 ± 1.9	< 0.001
Hematocrit, %	40.2 ± 5.1	37.6 ± 5.6	< 0.001
WBC Count, $10^3/\mu L$	7.5 ± 2.1	7.3 ± 2.5	0.330
Platelet count, $10^3/\mu L$	221.4 ± 54.3	192.9 ± 58.0	< 0.001
Activated partial thromboplastin time, seconds	29.7 ± 3.2	29.2 ± 3.2	0.060
Prothrombin time – INR, seconds	1.0 ± 0.1	1.0 ± 0.1	< 0.001
Total cholesterol, mg/dL	172.9 ± 40.1	152.1 ± 43.0	< 0.001
Blood urea nitrogen, mg/dL	14.3 ± 5.8	14.9 ± 5.6	0.250
Creatinine, mg/dL	0.9 ± 0.2	0.9 ± 0.2	0.041

INR: International normalized ratio; WBC: White blood cell count; SD: standard deviation; p-value were calculated with t-test for continuous variable and chi-square test for categorical variable.

Supplementary Table S3. Demographic table summarizes the differences in key clinical variables between the mortality within 3 year and non-mortality groups.

Variables	Non-mortality (n=1638)	Mortality (n=142)	p-value
Gender, n (%)			0.125
Male	957 (58.4%)	73 (51.4%)	
Female	681 (41.6%)	69 (48.6%)	
Age, n (%)			0.001
Under 60s	301 (18.4%)	10 (7.0%)	
60s and above	1337 (81.6%)	132 (93.0%)	
Vital sign, mean ± SD			
Body temperature, °C	36.7 ± 0.4	36.6 ± 0.4	0.645
Diastolic blood pressure, mmHg	82.0 ± 11.8	80.5 ± 11.6	0.126
Systolic blood pressure, mmHg	140.6 ± 20.7	137.2 ± 22.4	0.062
Pulse rate, bpm	76.6 ± 12.8	82.1 ± 15.4	< 0.001
Respiratory rate, bpm	19.3 ± 1.6	19.3 ± 1.9	0.877
Blood test, mean ± SD			
Hemoglobin, g/dL	13.7 ± 1.9	12.6 ± 1.8	< 0.001
Hematocrit, %	40.3 ± 5.1	37.5 ± 5.5	< 0.001
WBC Count, $10^3/\mu L$	7.5 ± 2.2	7.4 ± 2.5	0.573
Platelet count, $10^3/\mu L$	221.5 ± 54.4	194.2 ± 56.8	< 0.001
Activated partial thromboplastin time, seconds	29.7 ± 3.2	29.1 ± 3.3	0.023
Prothrombin time – INR, seconds	1.0 ± 0.1	1.0 ± 0.1	< 0.001
Total cholesterol, mg/dL	173.1 ± 40.2	151.6 ± 41.7	< 0.001
Blood urea nitrogen, mg/dL	14.3 ± 5.8	14.7 ± 5.6	0.448
Creatinine, mg/dL	0.9 ± 0.2	0.9 ± 0.2	0.053

INR: International normalized ratio; WBC: White blood cell count; SD: standard deviation; p-value were calculated with t-test for continuous variable and chi-square test for categorical variable.

Supplementary Table S4. AUC values and 95% confidence intervals for various models, assessing their overall predictive performance.

Model	Outcome	Input variable format	AUC	CI_lower	CI_upper
age0_model	outcome_1year	blood_binary	0.862	0.717	1.000
		blood_original	1.000	1.000	1.000
		vital_binary	0.708	0.531	0.885
		vital_binary_blood_binary	0.987	0.972	1.000
		vital_binary_blood_original	1.000	1.000	1.000
		vital_original	1.000	1.000	1.000
		vital_original_blood_binary	0.999	0.999	1.000
		vital_original_blood_original	1.000	1.000	1.000
age0_model	outcome_2year	blood_binary	0.862	0.717	1.000
		blood_original	1.000	1.000	1.000
		vital_binary	0.708	0.531	0.885
		vital_binary_blood_binary	0.987	0.972	1.000
		vital_binary_blood_original	1.000	1.000	1.000
		vital_original	1.000	1.000	1.000
		vital_original_blood_binary	0.999	0.999	1.000
		vital_original_blood_original	1.000	1.000	1.000
age0_model	outcome_3year	blood_binary	0.862	0.717	1.000

		blood_original	1.000	1.000	1.000
		vital_binary	0.708	0.531	0.885
		vital_binary_blood_binary	0.987	0.972	1.000
		vital_binary_blood_original	1.000	1.000	1.000
		vital_original	1.000	1.000	1.000
		vital_original_blood_binary	0.999	0.999	1.000
		vital_original_blood_original	1.000	1.000	1.000
age0_model	outcome_all	blood_binary	0.783	0.631	0.936
		blood_original	0.875	0.709	1.000
		vital_binary	0.669	0.513	0.824
		vital_binary_blood_binary	0.939	0.875	1.000
		vital_binary_blood_original	0.891	0.747	1.000
		vital_original	0.983	0.963	1.000
		vital_original_blood_binary	0.977	0.947	1.000
		vital_original_blood_original	0.910	0.791	1.000
age1_model	outcome_1year	blood_binary	0.708	0.654	0.762
		blood_original	0.888	0.838	0.938
		vital_binary	0.630	0.582	0.678
		vital_binary_blood_binary	0.834	0.785	0.882
		vital_binary_blood_original	0.920	0.881	0.959
		vital_original	0.916	0.879	0.953
		vital_original_blood_binary	0.939	0.913	0.966
		vital_original_blood_original	0.934	0.899	0.969
age1_model	outcome_2year	blood_binary	0.689	0.638	0.741
		blood_original	0.922	0.884	0.960
		vital_binary	0.604	0.561	0.647
		vital_binary_blood_binary	0.851	0.806	0.896
		vital_binary_blood_original	0.919	0.882	0.957
		vital_original	0.907	0.868	0.947
		vital_original_blood_binary	0.930	0.899	0.961
		vital_original_blood_original	0.957	0.937	0.977
age1_model	outcome_3year	blood_binary	0.686	0.636	0.736
		blood_original	0.886	0.842	0.929
		vital_binary	0.629	0.586	0.671
		vital_binary_blood_binary	0.836	0.791	0.882
		vital_binary_blood_original	0.912	0.875	0.948
		vital_original	0.917	0.884	0.950
		vital_original_blood_binary	0.928	0.896	0.959
		vital_original_blood_original	0.933	0.904	0.962
age1_model	outcome_all	blood_binary	0.680	0.632	0.728

		blood_original	0.929	0.895	0.965
		vital_binary	0.624	0.583	0.666
		vital_binary_blood_binary	0.850	0.808	0.893
		vital_binary_blood_original	0.941	0.911	0.971
		vital_original	0.932	0.903	0.961
		vital_original_blood_binary	0.962	0.942	0.981
		vital_original_blood_original	0.969	0.953	0.984
hist3_1_age0_model	outcome_1year	blood_binary	0.826	0.719	0.934
		blood_original	1.000	1.000	1.000
		vital_binary	1.000	1.000	1.000
		vital_binary_blood_binary	1.000	1.000	1.000
		vital_binary_blood_original	1.000	1.000	1.000
		vital_original	1.000	1.000	1.000
		vital_original_blood_binary	1.000	1.000	1.000
		vital_original_blood_original	1.000	1.000	1.000
hist3_1_age0_model	outcome_2year	blood_binary	0.826	0.719	0.934
		blood_original	1.000	1.000	1.000
		vital_binary	1.000	1.000	1.000
		vital_binary_blood_binary	1.000	1.000	1.000
		vital_binary_blood_original	1.000	1.000	1.000
		vital_original	1.000	1.000	1.000
		vital_original_blood_binary	1.000	1.000	1.000
		vital_original_blood_original	1.000	1.000	1.000
hist3_1_age0_model	outcome_3year	blood_binary	0.826	0.719	0.934
		blood_original	1.000	1.000	1.000
		vital_binary	1.000	1.000	1.000
		vital_binary_blood_binary	1.000	1.000	1.000
		vital_binary_blood_original	1.000	1.000	1.000
		vital_original	1.000	1.000	1.000
		vital_original_blood_binary	1.000	1.000	1.000
		vital_original_blood_original	1.000	1.000	1.000
hist3_1_age0_model	outcome_all	blood_binary	0.826	0.719	0.934
		blood_original	1.000	1.000	1.000
		vital_binary	1.000	1.000	1.000
		vital_binary_blood_binary	1.000	1.000	1.000
		vital_binary_blood_original	1.000	1.000	1.000
		vital_original	1.000	1.000	1.000
		vital_original_blood_binary	1.000	1.000	1.000
		vital_original_blood_original	1.000	1.000	1.000
hist3_1_age1_model	outcome_1year	blood_binary	0.709	0.633	0.786

		blood_original	0.843	0.764	0.922
		vital_binary	0.623	0.548	0.699
		vital_binary_blood_binary	0.828	0.759	0.896
		vital_binary_blood_original	0.905	0.849	0.961
		vital_original	0.924	0.870	0.977
		vital_original_blood_binary	0.926	0.884	0.967
		vital_original_blood_original	0.939	0.892	0.986
hist3_1_age1_model	outcome_2year	blood_binary	0.721	0.648	0.793
		blood_original	0.902	0.840	0.964
		vital_binary	0.651	0.581	0.721
		vital_binary_blood_binary	0.831	0.764	0.899
		vital_binary_blood_original	0.919	0.868	0.970
		vital_original	0.908	0.859	0.958
		vital_original_blood_binary	0.902	0.853	0.951
		vital_original_blood_original	0.954	0.921	0.987
hist3_1_age1_model	outcome_3year	blood_binary	0.704	0.629	0.779
		blood_original	0.869	0.796	0.942
		vital_binary	0.669	0.599	0.739
		vital_binary_blood_binary	0.865	0.804	0.927
		vital_binary_blood_original	0.920	0.872	0.968
		vital_original	0.935	0.891	0.978
		vital_original_blood_binary	0.932	0.893	0.972
		vital_original_blood_original	0.938	0.893	0.982
hist3_1_age1_model	outcome_all	blood_binary	0.698	0.624	0.772
		blood_original	0.870	0.803	0.937
		vital_binary	0.657	0.589	0.724
		vital_binary_blood_binary	0.852	0.794	0.911
		vital_binary_blood_original	0.916	0.871	0.961
		vital_original	0.926	0.881	0.970
		vital_original_blood_binary	0.936	0.900	0.972
		vital_original_blood_original	0.928	0.884	0.971
hist3_1_model	outcome_1year	blood_binary	0.734	0.661	0.806
		blood_original	0.909	0.844	0.975
		vital_binary	0.679	0.605	0.754
		vital_binary_blood_binary	0.865	0.803	0.927
		vital_binary_blood_original	0.927	0.872	0.982
		vital_original	0.935	0.886	0.983
		vital_original_blood_binary	0.955	0.923	0.986
		vital_original_blood_original	0.949	0.909	0.991
hist3_1_model	outcome_2year	blood_binary	0.731	0.659	0.802

		blood_original	0.922	0.870	0.974
		vital_binary	0.671	0.603	0.740
		vital_binary_blood_binary	0.870	0.808	0.933
		vital_binary_blood_original	0.931	0.883	0.979
		vital_original	0.928	0.879	0.977
		vital_original_blood_binary	0.943	0.905	0.981
		vital_original_blood_original	0.944	0.905	0.982
hist3_1_model	outcome_3year	blood_binary	0.719	0.648	0.789
		blood_original	0.895	0.831	0.959
		vital_binary	0.678	0.609	0.747
		vital_binary_blood_binary	0.870	0.813	0.928
		vital_binary_blood_original	0.934	0.894	0.974
		vital_original	0.931	0.889	0.974
		vital_original_blood_binary	0.935	0.897	0.972
		vital_original_blood_original	0.955	0.926	0.984
hist3_1_model	outcome_all	blood_binary	0.699	0.627	0.770
		blood_original	0.895	0.834	0.956
		vital_binary	0.670	0.604	0.736
		vital_binary_blood_binary	0.852	0.792	0.911
		vital_binary_blood_original	0.931	0.891	0.971
		vital_original	0.931	0.892	0.969
		vital_original_blood_binary	0.939	0.903	0.976
		vital_original_blood_original	0.964	0.943	0.986
hist4_1_age0_model	outcome_1year	blood_binary	0.860	0.706	1.000
		blood_original	1.000	1.000	1.000
		vital_binary	0.990	0.972	1.000
		vital_binary_blood_binary	0.985	0.958	1.000
		vital_binary_blood_original	1.000	1.000	1.000
		vital_original	1.000	1.000	1.000
		vital_original_blood_binary	1.000	1.000	1.000
		vital_original_blood_original	1.000	1.000	1.000
hist4_1_age0_model	outcome_2year	blood_binary	0.860	0.706	1.000
		blood_original	1.000	1.000	1.000
		vital_binary	0.990	0.972	1.000
		vital_binary_blood_binary	0.985	0.958	1.000
		vital_binary_blood_original	1.000	1.000	1.000
		vital_original	1.000	1.000	1.000
		vital_original_blood_binary	1.000	1.000	1.000
		vital_original_blood_original	1.000	1.000	1.000
hist4_1_age0_model	outcome_3year	blood_binary	0.860	0.706	1.000

		blood_original	1.000	1.000	1.000
		vital_binary	0.990	0.972	1.000
		vital_binary_blood_binary	0.985	0.958	1.000
		vital_binary_blood_original	1.000	1.000	1.000
		vital_original	1.000	1.000	1.000
		vital_original_blood_binary	1.000	1.000	1.000
		vital_original_blood_original	1.000	1.000	1.000
hist4_1_age0_model	outcome_all	blood_binary	0.860	0.706	1.000
		blood_original	1.000	1.000	1.000
		vital_binary	0.990	0.972	1.000
		vital_binary_blood_binary	0.985	0.958	1.000
		vital_binary_blood_original	1.000	1.000	1.000
		vital_original	1.000	1.000	1.000
		vital_original_blood_binary	1.000	1.000	1.000
		vital_original_blood_original	1.000	1.000	1.000
hist4_1_age1_model	outcome_1year	blood_binary	0.742	0.667	0.817
		blood_original	0.905	0.841	0.968
		vital_binary	0.663	0.589	0.737
		vital_binary_blood_binary	0.848	0.774	0.921
		vital_binary_blood_original	0.905	0.845	0.964
		vital_original	0.908	0.851	0.965
		vital_original_blood_binary	0.933	0.894	0.972
		vital_original_blood_original	0.944	0.906	0.982
hist4_1_age1_model	outcome_2year	blood_binary	0.706	0.633	0.778
		blood_original	0.898	0.835	0.959
		vital_binary	0.650	0.584	0.717
		vital_binary_blood_binary	0.85	0.798	0.911
		vital_binary_blood_original	0.911	0.853	0.969
		vital_original	0.906	0.856	0.956
		vital_original_blood_binary	0.919	0.871	0.967
		vital_original_blood_original	0.952	0.919	0.985
hist4_1_age1_model	outcome_3year	blood_binary	0.713	0.642	0.784
		blood_original	0.895	0.833	0.957
		vital_binary	0.662	0.593	0.731
		vital_binary_blood_binary	0.873	0.815	0.932
		vital_binary_blood_original	0.928	0.882	0.973
		vital_original	0.930	0.887	0.973
		vital_original_blood_binary	0.936	0.893	0.979
		vital_original_blood_original	0.962	0.935	0.989
hist4_1_age1_model	outcome_all	blood_binary	0.715	0.644	0.786

		blood_original	0.878	0.809	0.946
		vital_binary	0.652	0.585	0.719
		vital_binary_blood_binary	0.889	0.841	0.937
		vital_binary_blood_original	0.923	0.879	0.967
		vital_original	0.922	0.879	0.964
		vital_original_blood_binary	0.931	0.890	0.972
		vital_original_blood_original	0.938	0.897	0.979
hist4_1_model	outcome_1year	blood_binary	0.713	0.638	0.789
		blood_original	0.912	0.847	0.976
		vital_binary	0.662	0.588	0.736
		vital_binary_blood_binary	0.855	0.787	0.922
		vital_binary_blood_original	0.932	0.883	0.982
		vital_original	0.936	0.893	0.978
		vital_original_blood_binary	0.942	0.906	0.979
		vital_original_blood_original	0.962	0.933	0.991
hist4_1_model	outcome_2year	blood_binary	0.713	0.639	0.786
		blood_original	0.905	0.848	0.963
		vital_binary	0.633	0.567	0.698
		vital_binary_blood_binary	0.843	0.779	0.907
		vital_binary_blood_original	0.920	0.876	0.965
		vital_original	0.934	0.893	0.976
		vital_original_blood_binary	0.932	0.895	0.969
		vital_original_blood_original	0.965	0.939	0.989
hist4_1_model	outcome_3year	blood_binary	0.714	0.642	0.786
		blood_original	0.877	0.818	0.942
		vital_binary	0.664	0.597	0.730
		vital_binary_blood_binary	0.881	0.832	0.929
		vital_binary_blood_original	0.916	0.869	0.962
		vital_original	0.933	0.892	0.973
		vital_original_blood_binary	0.929	0.892	0.967
		vital_original_blood_original	0.947	0.917	0.976
hist4_1_model	outcome_all	blood_binary	0.690	0.621	0.759
		blood_original	0.889	0.831	0.947
		vital_binary	0.635	0.569	0.699
		vital_binary_blood_binary	0.832	0.771	0.893
		vital_binary_blood_original	0.932	0.894	0.969
		vital_original	0.907	0.861	0.952
		vital_original_blood_binary	0.942	0.909	0.975
		vital_original_blood_original	0.958	0.933	0.982
hist5_1_age0_model	outcome_1year	blood_binary	0.929	0.851	1.000

		blood_original	1.000	1.000	1.000
		vital_binary	0.990	0.970	1.000
		vital_binary_blood_binary	0.990	0.972	1.000
		vital_binary_blood_original	1.000	1.000	1.000
		vital_original	1.000	1.000	1.000
		vital_original_blood_binary	1.000	1.000	1.000
		vital_original_blood_original	1.000	1.000	1.000
hist5_1_age0_model	outcome_2year	blood_binary	0.929	0.851	1.000
		blood_original	1.000	1.000	1.000
		vital_binary	0.990	0.970	1.000
		vital_binary_blood_binary	0.990	0.972	1.000
		vital_binary_blood_original	1.000	1.000	1.000
		vital_original	1.000	1.000	1.000
		vital_original_blood_binary	1.000	1.000	1.000
		vital_original_blood_original	1.000	1.000	1.000
hist5_1_age0_model	outcome_3year	blood_binary	0.929	0.851	1.000
		blood_original	1.000	1.000	1.000
		vital_binary	0.990	0.970	1.000
		vital_binary_blood_binary	0.990	0.972	1.000
		vital_binary_blood_original	1.000	1.000	1.000
		vital_original	1.000	1.000	1.000
		vital_original_blood_binary	1.000	1.000	1.000
		vital_original_blood_original	1.000	1.000	1.000
hist5_1_age0_model	outcome_all	blood_binary	0.929	0.851	1.000
		blood_original	1.000	1.000	1.000
		vital_binary	0.990	0.970	1.000
		vital_binary_blood_binary	0.990	0.972	1.000
		vital_binary_blood_original	1.000	1.000	1.000
		vital_original	1.000	1.000	1.000
		vital_original_blood_binary	1.000	1.000	1.000
		vital_original_blood_original	1.000	1.000	1.000
hist5_1_age1_model	outcome_1year	blood_binary	0.709	0.634	0.786
		blood_original	0.892	0.824	0.959
		vital_binary	0.653	0.580	0.727
		vital_binary_blood_binary	0.843	0.775	0.911
		vital_binary_blood_original	0.916	0.860	0.972
		vital_original	0.934	0.886	0.982
		vital_original_blood_binary	0.938	0.903	0.973
		vital_original_blood_original	0.958	0.922	0.994
hist5_1_age1_model	outcome_2year	blood_binary	0.745	0.674	0.816

		blood_original	0.899	0.838	0.961
		vital_binary	0.656	0.588	0.724
		vital_binary_blood_binary	0.889	0.834	0.945
		vital_binary_blood_original	0.927	0.876	0.979
		vital_original	0.943	0.901]	0.984
		vital_original_blood_binary	0.956	0.925	0.986
		vital_original_blood_original	0.949	0.913	0.987
hist5_1_age1_model	outcome_3year	blood_binary	0.736	0.666	0.807
		blood_original	0.899	0.835	0.964
		vital_binary	0.662	0.596	0.728
		vital_binary_blood_binary	0.897	0.845	0.948
		vital_binary_blood_original	0.941	0.899	0.984
		vital_original	0.944	0.903	0.985
		vital_original_blood_binary	0.967	0.941	0.992
		vital_original_blood_original	0.965	0.936	0.995
hist5_1_age1_model	outcome_all	blood_binary	0.707	0.635	0.779
		blood_original	0.858	0.787	0.929
		vital_binary	0.654	0.589	0.719
		vital_binary_blood_binary	0.871	0.818	0.925
		vital_binary_blood_original	0.914	0.868	0.960
		vital_original	0.904	0.848	0.959
		vital_original_blood_binary	0.916	0.867	0.965
		vital_original_blood_original	0.923	0.878	0.969
hist5_1_model	outcome_1year	blood_binary	0.749	0.675	0.823
		blood_original	0.911	0.849	0.973
		vital_binary	0.687	0.614	0.759
		vital_binary_blood_binary	0.898	0.846	0.951
		vital_binary_blood_original	0.939	0.889	0.988
		vital_original	0.949	0.907	0.989
		vital_original_blood_binary	0.964	0.938	0.991
		vital_original_blood_original	0.965	0.934	0.995
hist5_1_model	outcome_2year	blood_binary	0.750	0.681	0.819
		blood_original	0.919	0.862	0.976
		vital_binary	0.676	0.609	0.743
		vital_binary_blood_binary	0.891	0.843	0.939
		vital_binary_blood_original	0.917	0.864	0.969
		vital_original	0.924	0.878	0.971
		vital_original_blood_binary	0.954	0.924	0.983
		vital_original_blood_original	0.946	0.907	0.986
hist5_1_model	outcome_3year	blood_binary	0.729	0.658	0.799

		blood_original	0.903	0.844	0.963
		vital_binary	0.672	0.607	0.738
		vital_binary_blood_binary	0.876	0.824	0.928
		vital_binary_blood_original	0.927	0.882	0.971
		vital_original	0.946	0.909	0.982
		vital_original_blood_binary	0.952	0.923	0.980
		vital_original_blood_original	0.965	0.940	0.989
hist5_1_model	outcome_all	blood_binary	0.719	0.651	0.788
		blood_original	0.913	0.858	0.968
		vital_binary	0.674	0.609	0.738
		vital_binary_blood_binary	0.882	0.831	0.933
		vital_binary_blood_original	0.937	0.898	0.977
		vital_original	0.933	0.890	0.975
		vital_original_blood_binary	0.964	0.939	0.989
		vital_original_blood_original	0.968	0.943	0.993
histTotal_1_age0_model	outcome_1year	blood_binary	0.854	0.655	1.000
		blood_original	1.000	1.000	1.000
		vital_binary	0.990	0.971	1.000
		vital_binary_blood_binary	0.995	0.984	1.000
		vital_binary_blood_original	1.000	1.000	1.000
		vital_original	1.000	1.000	1.000
		vital_original_blood_binary	1.000	1.000	1.000
		vital_original_blood_original	1.000	1.000	1.000
histTotal_1_age0_model	outcome_2year	blood_binary	0.854	0.655	1.000
		blood_original	1.000	1.000	1.000
		vital_binary	0.990	0.971	1.000
		vital_binary_blood_binary	0.995	0.984	1.000
		vital_binary_blood_original	1.000	1.000	1.000
		vital_original	1.000	1.000	1.000
		vital_original_blood_binary	1.000	1.000	1.000
		vital_original_blood_original	1.000	1.000	1.000
histTotal_1_age0_model	outcome_3year	blood_binary	0.854	0.655	1.000
		blood_original	1.000	1.000	1.000
		vital_binary	0.990	0.971	1.000
		vital_binary_blood_binary	0.995	0.984	1.000
		vital_binary_blood_original	1.000	1.000	1.000
		vital_original	1.000	1.000	1.000
		vital_original_blood_binary	1.000	1.000	1.000
		vital_original_blood_original	1.000	1.000	1.000
histTotal_1_age0_model	outcome_all	blood_binary	0.854	0.655	1.000

		blood_original	1.000	1.000	1.000
		vital_binary	0.990	0.971	1.000
		vital_binary_blood_binary	0.995	0.984	1.000
		vital_binary_blood_original	1.000	1.000	1.000
		vital_original	1.000	1.000	1.000
		vital_original_blood_binary	1.000	1.000	1.000
		vital_original_blood_original	1.000	1.000	1.000
histTotal_1_age1_model	outcome_1year	blood_binary	0.749	0.675	0.825
		blood_original	0.922	0.865	0.979
		vital_binary	0.628	0.556	0.699
		vital_binary_blood_binary	0.886	0.826	0.945
		vital_binary_blood_original	0.933	0.882	0.983
		vital_original	0.946	0.902	0.989
		vital_original_blood_binary	0.964	0.936	0.991
		vital_original_blood_original	0.968	0.939	0.997
histTotal_1_age1_model	outcome_2year	blood_binary	0.763	0.696	0.831
		blood_original	0.927	0.876	0.978
		vital_binary	0.638	0.571	0.705
		vital_binary_blood_binary	0.905	0.861	0.949
		vital_binary_blood_original	0.942	0.897	0.987
		vital_original	0.946	0.905	0.987
		vital_original_blood_binary	0.969	0.946	0.994
		vital_original_blood_original	0.963	0.931	0.995
histTotal_1_age1_model	outcome_3year	blood_binary	0.728	0.656	0.800
		blood_original	0.888	0.824	0.953
		vital_binary	0.647	0.582	0.712
		vital_binary_blood_binary	0.874	0.819	0.929
		vital_binary_blood_original	0.922	0.879	0.964
		vital_original	0.927	0.882	0.973
		vital_original_blood_binary	0.951	0.921	0.982
		vital_original_blood_original	0.962	0.937	0.986
histTotal_1_age1_model	outcome_all	blood_binary	0.718	0.649	0.786
		blood_original	0.883	0.822	0.944
		vital_binary	0.648	0.585	0.712
		vital_binary_blood_binary	0.893	0.849	0.937
		vital_binary_blood_original	0.927	0.884	0.971
		vital_original	0.890	0.836	0.945
		vital_original_blood_binary	0.938	0.899	0.979
		vital_original_blood_original	0.944	0.915	0.974
histTotal_model	outcome_1year	blood_binary	0.772	0.704	0.839

		blood_original	0.918	0.860	0.978
		vital_binary	0.674	0.602	0.745
		vital_binary_blood_binary	0.895	0.842	0.949
		vital_binary_blood_original	0.932	0.884	0.980
		vital_original	0.939	0.895	0.983
		vital_original_blood_binary	0.966	0.941	0.991
		vital_original_blood_original	0.968	0.942	0.995
histTotal_model	outcome_2year	blood_binary	0.770	0.704	0.837
		blood_original	0.933	0.885	0.981
		vital_binary	0.656	0.590	0.723
		vital_binary_blood_binary	0.901	0.854	0.948
		vital_binary_blood_original	0.947	0.907	0.986
		vital_original	0.943	0.905	0.981
		vital_original_blood_binary	0.963	0.936	0.989
		vital_original_blood_original	0.969	0.944	0.994
histTotal_model	outcome_3year	blood_binary	0.751	0.684	0.817
		blood_original	0.916	0.862	0.970
		vital_binary	0.669	0.604	0.734
		vital_binary_blood_binary	0.904	0.857	0.951
		vital_binary_blood_original	0.947	0.910	0.984
		vital_original	0.942	0.904	0.981
		vital_original_blood_binary	0.970	0.951	0.989
		vital_original_blood_original	0.957	0.927	0.988
histTotal_model	outcome_all	blood_binary	0.741	0.674	0.807
		blood_original	0.909	0.856	0.963
		vital_binary	0.658	0.595	0.722
		vital_binary_blood_binary	0.891	0.844	0.937
		vital_binary_blood_original	0.937	0.898	0.975
		vital_original	0.944	0.909	0.979
		vital_original_blood_binary	0.956	0.928	0.983
		vital_original_blood_original	0.962	0.935	0.989
totalData_model	outcome_1year	blood_binary	0.705	0.652	0.758
		blood_original	0.928	0.889	0.967
		vital_binary	0.621	0.576	0.667
		vital_binary_blood_binary	0.844	0.798	0.890
		vital_binary_blood_original	0.941	0.908	0.974
		vital_original	0.932	0.901	0.962
		vital_original_blood_binary	0.960	0.941	0.979
		vital_original_blood_original	0.974	0.958	0.989
totalData_model	outcome_2year	blood_binary	0.709	0.659	0.758

		blood_original	0.943	0.909	0.976
		vital_binary	0.616	0.573	0.658
		vital_binary_blood_binary	0.862	0.820	0.904
		vital_binary_blood_original	0.948	0.917	0.979
		vital_original	0.943	0.913	0.972
		vital_original_blood_binary	0.963	0.944	0.983
		vital_original_blood_original	0.971	0.953	0.988
totalData_model	outcome_3year	blood_binary	0.684	0.636	0.732
		blood_original	0.913	0.876	0.949
		vital_binary	0.627	0.586	0.669
		vital_binary_blood_binary	0.854	0.815	0.892
		vital_binary_blood_original	0.925	0.893	0.957
		vital_original	0.936	0.909	0.963
		vital_original_blood_binary	0.949	0.926	0.972
		vital_original_blood_original	0.955	0.937	0.973
totalData_model	outcome_all	blood_binary	0.698	0.652	0.744
		blood_original	0.934	0.901	0.966
		vital_binary	0.621	0.580	0.661
		vital_binary_blood_binary	0.851	0.811	0.891
		vital_binary_blood_original	0.943	0.914	0.972
		vital_original	0.945	0.921	0.969
		vital_original_blood_binary	0.967	0.951	0.984
		vital_original_blood_original	0.975	0.963	0.987

age0: Age was coded as 0 for individuals under 60. totalData: The complete dataset including all variables. age1: Age was binarized as 1 for individuals aged 60 and above. hist5_1: History variable was binarized into 1 for the top 5. Hist4_1: History variable was binarized into 1 for the top 4. hist3_1: History variable was binarized into 1 for the top 3. histTotal: History variable was binarized as 1 if any of the considered conditions were present.

Supplementary Table S5. Top-ranked feature importance result for each model. For each combination of subgroup, outcome, and input variable format, only the single variable with the highest feature importance is shown as a concise summary; the full set of feature importance values for all variables is not presented in this table.

Model	Outcome	Input variable format	Feature	Importance
age0_model	outcome_1year	blood_binary	WBC_class	2.943
		blood_original	WBC	2.677
		vital_binary	BT_class	1.914
		vital_binary_blood_binary	WBC_class	2.864
		vital_binary_blood_original	WBC	2.173
		vital_original	DBP	4.957
		vital_original_blood_binary	DBP	2.764
		vital_original_blood_original	WBC	1.967
age0_model	outcome_2year	blood_binary	WBC_class	2.943
		blood_original	WBC	2.677
		vital_binary	BT_class	1.914

		vital_binary_blood_binary	WBC_class	2.864
		vital_binary_blood_original	WBC	2.173
		vital_original	DBP	4.957
		vital_original_blood_binary	DBP	2.764
		vital_original_blood_original	WBC	1.967
age0_model	outcome_3year	blood_binary	WBC_class	2.943
		blood_original	WBC	2.677
		vital_binary	BT_class	1.914
		vital_binary_blood_binary	WBC_class	2.864
		vital_binary_blood_original	WBC	2.173
		vital_original	DBP	4.957
		vital_original_blood_binary	DBP	2.764
		vital_original_blood_original	WBC	1.967
age0_model	outcome_all	blood_binary	WBC_class	3.029
		blood_original	WBC	3.578
		vital_binary	BT_class	2.410
		vital_binary_blood_binary	WBC_class	2.899
		vital_binary_blood_original	WBC	2.645
		vital_original	SBP	6.295
		vital_original_blood_binary	SBP	6.030
		vital_original_blood_original	aPTT	2.020
age1_model	outcome_1year	blood_binary	WBC_class	9.354
		blood_original	PC	25.068
		vital_binary	PR_class	5.489
		vital_binary_blood_binary	DBP_class	12.721
		vital_binary_blood_original	PC	20.682
		vital_original	PR	49.392
		vital_original_blood_binary	PR	22.908
		vital_original_blood_original	PR	19.106
age1_model	outcome_2year	blood_binary	WBC_class	12.819
		blood_original	PC	28.076
		vital_binary	PR_class	5.721
		vital_binary_blood_binary	gender	15.240
		vital_binary_blood_original	PC	21.195
		vital_original	PR	55.851
		vital_original_blood_binary	PR	44.324
		vital_original_blood_original	PR	20.659
age1_model	outcome_3year	blood_binary	WBC_class	12.801
		blood_original	PC	29.802
		vital_binary	PR_class	6.675

		vital_binary_blood_binary	TC_class	18.271
		vital_binary_blood_original	PC	23.481
		vital_original	PR	65.693
		vital_original_blood_binary	PR	49.829
		vital_original_blood_original	PR	23.645
age1_model	outcome_all	blood_binary	WBC_class	13.827
		blood_original	PC	30.843
		vital_binary	PR_class	6.799
		vital_binary_blood_binary	gender	19.271
		vital_binary_blood_original	PC	24.198
		vital_original	PR	65.834
		vital_original_blood_binary	PR	52.154
		vital_original_blood_original	PR	23.937
hist3_1_age0_model	outcome_1year	blood_binary	BUN_class	0.483
		blood_original	TC	0.972
		vital_binary	RR_class	1.346
		vital_binary_blood_binary	RR_class	0.741
		vital_binary_blood_original	aPTT	0.790
		vital_original	RR	1.833
		vital_original_blood_binary	RR	1.029
		vital_original_blood_original	TC	0.684
hist3_1_age0_model	outcome_2year	blood_binary	BUN_class	0.483
		blood_original	TC	0.972
		vital_binary	RR_class	1.346
		vital_binary_blood_binary	RR_class	0.741
		vital_binary_blood_original	aPTT	0.790
		vital_original	RR	1.833
		vital_original_blood_binary	RR	1.029
		vital_original_blood_original	TC	0.684
hist3_1_age0_model	outcome_3year	blood_binary	BUN_class	0.483
		blood_original	TC	0.972
		vital_binary	RR_class	1.346
		vital_binary_blood_binary	RR_class	0.741
		vital_binary_blood_original	aPTT	0.790
		vital_original	RR	1.833
		vital_original_blood_binary	RR	1.029
		vital_original_blood_original	TC	0.684
hist3_1_age0_model	outcome_all	blood_binary	BUN_class	0.483
		blood_original	TC	0.972
		vital_binary	RR_class	1.346

		vital_binary_blood_binary	RR_class	0.741
		vital_binary_blood_original	aPTT	0.790
		vital_original	RR	1.833
		vital_original_blood_binary	RR	1.029
		vital_original_blood_original	TC	0.684
hist3_1_age1_model	outcome_1year	blood_binary	gender	5.824
		blood_original	PC	12.789
		vital_binary	DBP_class	5.637
		vital_binary_blood_binary	DBP_class	7.561
		vital_binary_blood_original	PC	10.932
		vital_original	PR	23.816
		vital_original_blood_binary	PR	14.784
		vital_original_blood_original	PR	12.179
hist3_1_age1_model	outcome_2year	blood_binary	gender	8.644
		blood_original	PC	15.641
		vital_binary	DBP_class	5.793
		vital_binary_blood_binary	gender	7.765
		vital_binary_blood_original	PC	12.734
		vital_original	PR	25.371
		vital_original_blood_binary	PR	24.896
		vital_original_blood_original	PR	11.929
hist3_1_age1_model	outcome_3year	blood_binary	gender	8.274
		blood_original	PC	15.383
		vital_binary	PR_class	3.886
		vital_binary_blood_binary	gender	7.969
		vital_binary_blood_original	HMT	12.492
		vital_original	PR	29.745
		vital_original_blood_binary	PR	17.061
		vital_original_blood_original	PR	13.829
hist3_1_age1_model	outcome_all	blood_binary	WBC_class	6.984
		blood_original	PC	15.535
		vital_binary	DBP_class	5.979
		vital_binary_blood_binary	DBP_class	8.003
		vital_binary_blood_original	PC	12.867
		vital_original	PR	33.274
		vital_original_blood_binary	PR	30.241
		vital_original_blood_original	PR	14.260
hist3_1_model	outcome_1year	blood_binary	gender	7.649
		blood_original	PC	13.628
		vital_binary	DBP_class	6.435

		vital_binary_blood_binary	DBP_class	8.014
		vital_binary_blood_original	PC	10.939
		vital_original	PR	22.269
		vital_original_blood_binary	PR	14.315
		vital_original_blood_original	PR	9.982
hist3_1_model	outcome_2year	blood_binary	gender	8.385
		blood_original	PC	15.234
		vital_binary	DBP_class	7.637
		vital_binary_blood_binary	DBP_class	9.339
		vital_binary_blood_original	PC	13.696
		vital_original	PR	23.980
		vital_original_blood_binary	PR	14.648
		vital_original_blood_original	PC	10.975
hist3_1_model	outcome_3year	blood_binary	gender	8.322
		blood_original	PC	15.984
		vital_binary	DBP_class	6.308
		vital_binary_blood_binary	DBP_class	8.542
		vital_binary_blood_original	PC	12.924
		vital_original	PR	26.022
		vital_original_blood_binary	PR	15.63
		vital_original_blood_original	PR	11.864
hist3_1_model	outcome_all	blood_binary	gender	8.306
		blood_original	PC	16.214
		vital_binary	DBP_class	7.963
		vital_binary_blood_binary	DBP_class	10.054
		vital_binary_blood_original	PC	13.284
		vital_original	PR	27.757
		vital_original_blood_binary	SBP	23.247
		vital_original_blood_original	PR	11.865
hist4_1_age0_model	outcome_1year	blood_binary	PC_class	2.813
		blood_original	aPTT	1.574
		vital_binary	RR_class	1.167
		vital_binary_blood_binary	PC_class	0.955
		vital_binary_blood_original	aPTT	1.579
		vital_original	SBP	2.441
		vital_original_blood_binary	SBP	1.204
		vital_original_blood_original	aPTT	1.236
hist4_1_age0_model	outcome_2year	blood_binary	PC_class	2.813
		blood_original	aPTT	1.574
		vital_binary	RR_class	1.167

		vital_binary_blood_binary	PC_class	0.955
		vital_binary_blood_original	aPTT	1.579
		vital_original	SBP	2.441
		vital_original_blood_binary	SBP	1.204
		vital_original_blood_original	aPTT	1.236
hist4_1_age0_model	outcome_3year	blood_binary	PC_class	2.813
		blood_original	aPTT	1.574
		vital_binary	RR_class	1.167
		vital_binary_blood_binary	PC_class	0.955
		vital_binary_blood_original	aPTT	1.579
		vital_original	SBP	2.441
		vital_original_blood_binary	SBP	1.204
		vital_original_blood_original	aPTT	1.236
hist4_1_age0_model	outcome_all	blood_binary	PC_class	2.813
		blood_original	aPTT	1.574
		vital_binary	RR_class	1.167
		vital_binary_blood_binary	PC_class	0.955
		vital_binary_blood_original	aPTT	1.579
		vital_original	SBP	2.441
		vital_original_blood_binary	SBP	1.204
		vital_original_blood_original	aPTT	1.236
hist4_1_age1_model	outcome_1year	blood_binary	gender	7.420
		blood_original	PC	13.816
		vital_binary	PR_class	4.597
		vital_binary_blood_binary	DBP_class	8.759
		vital_binary_blood_original	PC	14.676
		vital_original	PR	24.552
		vital_original_blood_binary	PR	15.162
		vital_original_blood_original	PR	11.672
hist4_1_age1_model	outcome_2year	blood_binary	WBC_class	7.305
		blood_original	PC	15.594
		vital_binary	DBP_class	6.706
		vital_binary_blood_binary	DBP_class	8.043
		vital_binary_blood_original	HMT	12.774
		vital_original	PR	26.221
		vital_original_blood_binary	PR	15.662
		vital_original_blood_original	PR	11.667
hist4_1_age1_model	outcome_3year	blood_binary	WBC_class	7.073
		blood_original	PC	15.760
		vital_binary	PR_class	3.551

		vital_binary_blood_binary	TC_class	9.928
		vital_binary_blood_original	PC	12.622
		vital_original	PR	30.835
		vital_original_blood_binary	PR	29.029
		vital_original_blood_original	PR	13.204
hist4_1_age1_model	outcome_all	blood_binary	WBC_class	8.649
		blood_original	PC	16.431
		vital_binary	PR_class	3.704
		vital_binary_blood_binary	TC_class	9.296
		vital_binary_blood_original	PC	12.986
		vital_original	PR	30.004
		vital_original_blood_binary	PR	18.046
		vital_original_blood_original	PR	13.585
hist4_1_model	outcome_1year	blood_binary	gender	6.973
		blood_original	PC	14.260
		vital_binary	PR_class	3.391
		vital_binary_blood_binary	DBP_class	9.897
		vital_binary_blood_original	PC	11.843
		vital_original	PR	22.928
		vital_original_blood_binary	PR	14.446
		vital_original_blood_original	PC	9.890
hist4_1_model	outcome_2year	blood_binary	WBC_class	7.647
		blood_original	PC	16.395
		vital_binary	DBP_class	6.7340
		vital_binary_blood_binary	TC_class	9.478
		vital_binary_blood_original	PC	18.406
		vital_original	SBP	25.751
		vital_original_blood_binary	SBP	21.641
		vital_original_blood_original	PC	11.872
hist4_1_model	outcome_3year	blood_binary	WBC_class	7.954
		blood_original	PC	17.209
		vital_binary	DBP_class	7.494
		vital_binary_blood_binary	TC_class	10.646
		vital_binary_blood_original	PC	14.179
		vital_original	PR	27.782
		vital_original_blood_binary	PR	16.123
		vital_original_blood_original	PR	11.589
hist4_1_model	outcome_all	blood_binary	WBC_class	8.961
		blood_original	PC	16.554
		vital_binary	DBP_class	5.614

		vital_binary_blood_binary	DBP_class	10.039
		vital_binary_blood_original	PC	14.612
		vital_original	PR	27.865
		vital_original_blood_binary	PR	23.998
		vital_original_blood_original	PC	11.732
hist5_1_age0_model	outcome_1year	blood_binary	PC_class	5.903
		blood_original	WBC	1.989
		vital_binary	SBP_class	1.749
		vital_binary_blood_binary	PC_class	1.758
		vital_binary_blood_original	aPTT	1.599
		vital_original	SBP	3.911
		vital_original_blood_binary	SBP	1.849
		vital_original_blood_original	WBC	1.422
hist5_1_age0_model	outcome_2year	blood_binary	PC_class	5.903
		blood_original	WBC	1.989
		vital_binary	SBP_class	1.749
		vital_binary_blood_binary	PC_class	1.758
		vital_binary_blood_original	aPTT	1.599
		vital_original	SBP	3.911
		vital_original_blood_binary	SBP	1.849
		vital_original_blood_original	WBC	1.422
hist5_1_age0_model	outcome_3year	blood_binary	PC_class	5.903
		blood_original	WBC	1.989
		vital_binary	SBP_class	1.749
		vital_binary_blood_binary	PC_class	1.758
		vital_binary_blood_original	aPTT	1.599
		vital_original	SBP	3.911
		vital_original_blood_binary	SBP	1.849
		vital_original_blood_original	WBC	1.422
hist5_1_age0_model	outcome_all	blood_binary	PC_class	5.903
		blood_original	WBC	1.989
		vital_binary	SBP_class	1.749
		vital_binary_blood_binary	PC_class	1.758
		vital_binary_blood_original	aPTT	1.599
		vital_original	SBP	3.911
		vital_original_blood_binary	SBP	1.849
		vital_original_blood_original	WBC	1.422
hist5_1_age1_model	outcome_1year	blood_binary	gender	6.046
		blood_original	PC	13.666
		vital_binary	PR_class	3.982

		vital_binary_blood_binary	DBP_class	8.488
		vital_binary_blood_original	PC	15.201
		vital_original	PR	24.914
		vital_original_blood_binary	PR	14.977
		vital_original_blood_original	PR	11.509
hist5_1_age1_model	outcome_2year	blood_binary	gender	8.637
		blood_original	PC	19.0522
		vital_binary	DBP_class	5.346
		vital_binary_blood_binary	gender	8.947
		vital_binary_blood_original	PC	17.266
		vital_original	PR	27.314
		vital_original_blood_binary	PR	24.077
		vital_original_blood_original	PR	11.305
hist5_1_age1_model	outcome_3year	blood_binary	gender	8.275
		blood_original	PC	15.804
		vital_binary	DBP_class	6.938
		vital_binary_blood_binary	TC_class	9.898
		vital_binary_blood_original	PC	12.759
		vital_original	PR	30.569
		vital_original_blood_binary	PR	28.629
		vital_original_blood_original	PR	13.867
hist5_1_age1_model	outcome_all	blood_binary	WBC_class	7.585
		blood_original	PC	20.496
		vital_binary	DBP_class	6.911
		vital_binary_blood_binary	TC_class	9.318
		vital_binary_blood_original	PC	13.859
		vital_original	PR	34.147
		vital_original_blood_binary	PR	29.765
		vital_original_blood_original	PR	14.279
hist5_1_model	outcome_1year	blood_binary	gender	8.565
		blood_original	PC	16.355
		vital_binary	DBP_class	4.999
		vital_binary_blood_binary	DBP_class	9.187
		vital_binary_blood_original	PC	13.231
		vital_original	PR	24.121
		vital_original_blood_binary	SBP	20.532
		vital_original_blood_original	PC	11.544
hist5_1_model	outcome_2year	blood_binary	gender	9.748
		blood_original	PC	17.798
		vital_binary	DBP_class	7.858

		vital_binary_blood_binary	DBP_class	8.929
		vital_binary_blood_original	PC	21.117
		vital_original	SBP	27.717
		vital_original_blood_binary	PR	15.169
		vital_original_blood_original	PC	12.596
hist5_1_model	outcome_3year	blood_binary	WBC_class	8.073
		blood_original	PC	18.216
		vital_binary	DBP_class	6.167
		vital_binary_blood_binary	DBP_class	9.623
		vital_binary_blood_original	PC	15.145
		vital_original	SBP	28.036
		vital_original_blood_binary	PR	16.293
		vital_original_blood_original	PC	12.857
hist5_1_model	outcome_all	blood_binary	WBC_class	8.171
		blood_original	PC	18.119
		vital_binary	DBP_class	8.138
		vital_binary_blood_binary	DBP_class	10.059
		vital_binary_blood_original	PC	15.651
		vital_original	PR	28.833
		vital_original_blood_binary	SBP	27.151
		vital_original_blood_original	PC	13.090
histTotal_1_age0_model	outcome_1year	blood_binary	PC_class	5.712
		blood_original	aPTT	1.996
		vital_binary	SBP_class	1.787
		vital_binary_blood_binary	PC_class	1.553
		vital_binary_blood_original	WBC	1.478
		vital_original	SBP	3.865
		vital_original_blood_binary	SBP	1.908
		vital_original_blood_original	aPTT	1.430
histTotal_1_age0_model	outcome_2year	blood_binary	PC_class	5.712
		blood_original	aPTT	1.996
		vital_binary	SBP_class	1.787
		vital_binary_blood_binary	PC_class	1.553
		vital_binary_blood_original	WBC	1.478
		vital_original	SBP	3.865
		vital_original_blood_binary	SBP	1.908
		vital_original_blood_original	aPTT	1.430
histTotal_1_age0_model	outcome_3year	blood_binary	PC_class	5.712
		blood_original	aPTT	1.996
		vital_binary	SBP_class	1.787

		vital_binary_blood_binary	PC_class	1.553
		vital_binary_blood_original	WBC	1.478
		vital_original	SBP	3.865
		vital_original_blood_binary	SBP	1.908
		vital_original_blood_original	aPTT	1.430
histTotal_1_age0_model	outcome_all	blood_binary	PC_class	5.712
		blood_original	aPTT	1.996
		vital_binary	SBP_class	1.787
		vital_binary_blood_binary	PC_class	1.553
		vital_binary_blood_original	WBC	1.478
		vital_original	SBP	3.865
		vital_original_blood_binary	SBP	1.908
		vital_original_blood_original	aPTT	1.430
histTotal_1_age1_model	outcome_1year	blood_binary	gender	7.789
		blood_original	PC	13.687
		vital_binary	PR_class	4.483
		vital_binary_blood_binary	DBP_class	9.630
		vital_binary_blood_original	PC	14.914
		vital_original	PR	27.045
		vital_original_blood_binary	PR	15.835
		vital_original_blood_original	PR	12.664
histTotal_1_age1_model	outcome_2year	blood_binary	gender	8.805
		blood_original	PC	15.402
		vital_binary	DBP_class	5.156
		vital_binary_blood_binary	gender	9.967
		vital_binary_blood_original	HMT	12.529
		vital_original	PR	28.923
		vital_original_blood_binary	PR	16.696
		vital_original_blood_original	PR	12.686
histTotal_1_age1_model	outcome_3year	blood_binary	WBC_class	7.523
		blood_original	PC	15.741
		vital_binary	DBP_class	7.039
		vital_binary_blood_binary	gender	10.534
		vital_binary_blood_original	PC	13.471
		vital_original	PR	36.643
		vital_original_blood_binary	PR	18.744
		vital_original_blood_original	PR	14.737
histTotal_1_age1_model	outcome_all	blood_binary	WBC_class	8.986
		blood_original	PC	16.296
		vital_binary	DBP_class	7.208

		vital_binary_blood_binary	gender	9.228
		vital_binary_blood_original	HMT	13.357
		vital_original	PR	37.717
		vital_original_blood_binary	PR	19.139
		vital_original_blood_original	PR	14.496
histTotal_model	outcome_1year	blood_binary	gender	8.952
		blood_original	PC	15.703
		vital_binary	DBP_class	4.730
		vital_binary_blood_binary	DBP_class	10.911
		vital_binary_blood_original	PC	12.811
		vital_original	PR	25.909
		vital_original_blood_binary	PR	14.957
		vital_original_blood_original	PC	12.013
histTotal_model	outcome_2year	blood_binary	gender	9.954
		blood_original	PC	17.489
		vital_binary	RR_class	3.445
		vital_binary_blood_binary	gender	11.111
		vital_binary_blood_original	PC	14.157
		vital_original	PR	27.368
		vital_original_blood_binary	PR	16.046
		vital_original_blood_original	PC	13.075
histTotal_model	outcome_3year	blood_binary	WBC_class	9.852
		blood_original	PC	17.533
		vital_binary	DBP_class	6.023
		vital_binary_blood_binary	gender	11.144
		vital_binary_blood_original	PC	14.373
		vital_original	PR	32.204
		vital_original_blood_binary	PR	17.234
		vital_original_blood_original	PC	12.783
histTotal_model	outcome_all	blood_binary	WBC_class	8.474
		blood_original	PC	20.989
		vital_binary	DBP_class	8.106
		vital_binary_blood_binary	DBP_class	10.146
		vital_binary_blood_original	PC	15.275
		vital_original	PR	31.301
		vital_original_blood_binary	PR	17.643
		vital_original_blood_original	PC	13.221
totalData_model	outcome_1year	blood_binary	WBC_class	13.029
		blood_original	PC	27.899
		vital_binary	SBP_class	6.384

		vital_binary_blood_binary	WBC_class	18.357
		vital_binary_blood_original	PC	22.645
		vital_original	PR	44.640
		vital_original_blood_binary	PR	39.787
		vital_original_blood_original	PC	19.717
totalData_model	outcome_2year	blood_binary	WBC_class	14.852
		blood_original	PC	30.612
		vital_binary	PR_class	5.129
		vital_binary_blood_binary	WBC_class	18.959
		vital_binary_blood_original	HMT	24.949
		vital_original	PR	51.379
		vital_original_blood_binary	PR	44.672
		vital_original_blood_original	PC	21.170
totalData_model	outcome_3year	blood_binary	WBC_class	13.300
		blood_original	PC	32.695
		vital_binary	SBP_class	5.912
		vital_binary_blood_binary	TC_class	21.160
		vital_binary_blood_original	TC	25.992
		vital_original	PR	57.732
		vital_original_blood_binary	PR	49.669
		vital_original_blood_original	PR	23.349
totalData_model	outcome_all	blood_binary	WBC_class	14.418
		blood_original	PC	34.369
		vital_binary	RR_class	6.140
		vital_binary_blood_binary	WBC_class	22.304
		vital_binary_blood_original	PC	28.123
		vital_original	PR	59.383
		vital_original_blood_binary	PR	51.878
		vital_original_blood_original	TC	23.746

Due to the large number of models and variables, only the variable with the highest feature importance value is reported for each combination of subgroup, outcome, and input variable format. The corresponding feature importance value for the selected variable is also presented. Age0: Age was coded as 0 for individuals under 60. totalData: The complete dataset including all variables. age1: Age was binarized as 1 for individuals aged 60 and above. hist5_1: History variable was binarized into 1 for the top 5. Hist4_1: History variable was binarized into 1 for the top 4. hist3_1: History variable was binarized into 1 for the top 3. histTotal: History variable was binarized as 1 if any of the considered conditions were present. Variables with the ‘class’ indicate binarized versions of the original variables. PR: Pulse rate; PC: Platelet count; DBP: Diastolic blood pressure; WBC: White blood cell;

Supplementary Table S6. Number of patients in subgroups defined by age and medical history.

Category	Subgroup	Total (n)
Age	age0	311
	age1	1469
History	hist5_1	583
	hist4_1	562
	hist3_1	538
	histTotal	638
Age + History (age<60)	hist5_1_age0	75
	hist4_1_age0	72
	hist3_1_age0	61
	histTotal_1_age0	77
Age + History (age≥60)	hist5_1_age1	508
	hist4_1_age1	490
	hist3_1_age1	477
	histTotal_1_age1	561

Subgroups were defined based on age (<60 or ≥60) and the presence of medical history conditions. History subgroups include patients with at least one of the top 3, top 4, top 5 conditions, or any history condition (histTotal). Age + History subgroups combine age and history stratification. age0: Age was coded as 0 for individuals under 60. age1: Age was binarized as 1 for individuals aged 60 and above. hist5_1: History variable was binarized into 1 for the top 5. Hist4_1: History variable was binarized into 1 for the top 4. hist3_1: History variable was binarized into 1 for the top 3. histTotal: History variable was binarized as 1 if any of the considered conditions were present.
